# Supplementary material for: Protective Role of Hepassocin against Hepatic Endoplasmic Reticulum Stress in Mice
Source: Int J Mol Sci. 2022 Nov 1;23(21):13325. doi: 10.3390/ijms232113325 (PMC9658083; doi:10.3390/ijms232113325)
Supplement: Supplementary file 1 [file ijms-23-13325-s001.zip › Supplementary figure legend.pdf]

Supplementary figure legend

Supplementary figure S1

(A) HEK293 cells or (B) HeLa cells were treated with 100 or 400ng/ml HPS for 2h followed by dealing with 4μg/ml TM for 12h as indicated. Quantitative real-time-PCR analysis of mRNA expression levels of *Grp78*, *Chop*, *Derl3*, *Hrd1*, *Herpud1* and *Erdj4* as indicated. Gene expression levels were normalized to *Tbp* mRNA levels. Data are represented as mean±SEM of triplicate experiments.

Supplementary figure S2

C57BL/6J wild-type (WT) and HPS-deficient (KO) male mice at 8 weeks of age were injected i.p. with buffer (ctrl) or TM (1mg/kg body weight). (A) Serum BUN levels of the mice at 24h after TM injection (n=4-5). (B) Quantitative real-time-PCR analysis of mRNA expression levels of *Grp78*, *Chop*, *Atf4*, *Atf6*, *Hsp40*, *Hsp90*, *Perk*, *Wfs1*, *Xbp1*, *Unspliced-Xbp1* and *Spliced Xbp1* in mice kidney as indicated. Gene expression levels were normalized to *Tbp* mRNA levels. Data are represented as mean±SD of triplicate experiments.

Supplementary figure S3

C57BL/6J wild-type (WT) and HPS-deficient (KO) male mice at 8 weeks of age were injected i.p. with buffer (ctrl) or TM (1mg/kg body weight). (A) Quantitative real-time-PCR analysis of mRNA expression levels of *Grp78*, *Chop*, *Erdj4 and Herpud1* in mice liver at 6 h after TM injection. Gene expression levels were normalized to *Tbp* mRNA levels (n=3). (B) Serum triglyceride, TCHO and ALT levels of the mice at 6 h after TM injection (n=5). Data are represented as mean±SD of triplicate experiments.
