# Supplementary material for: Protective Role of Hepassocin against Hepatic Endoplasmic Reticulum Stress in Mice
Source: Int J Mol Sci. 2022 Nov 1;23(21):13325. doi: 10.3390/ijms232113325 (PMC9658083; doi:10.3390/ijms232113325)
Supplement: Supplementary file 1 [file ijms-23-13325-s001.zip › Supplementary Table S1.pdf]

Supplementary Table S1: Antibodies used for immunoblot

Protein name	UniProtKB No.	Antibody	Identifier	Dilution
78 kDa glucose-regulated protein (GRP78)	P20029	Rabbit polyclonal GRP78 antibody	Abcam, ab21685	1:400
C/EBP-homologous protein (CHOP)	P35639	mouse monoclonal CHOP antibody	Abcam, ab11419	1:400
PRKR-like endoplasmic reticulum kinase (PERK)	Q9Z2B5	rabbit monoclonal PERK antibody	Abcam, ab229912	1:400
Eukaryotic translation initiation factor 2 subunit 1 (eIF2α)	Q6ZWX6	rabbit monoclonal eIF2α antibody	Abcam, ab169528	1:400
Cyclic AMP-dependent transcription factor ATF-4 (ATF4)	Q06507	rabbit polyclonal ATF4 antibody	Abcam, ab23760	1:400
Phospho-PERK (P-PERK)	N/A	Rabbit monoclonal PERK (phospho T980) antibody	Cell Signaling Technology, 3179	1:400
Phospho-eIF2α (P-eIF2α)	N/A	rabbit polyclonal EIF2α (phospho S51) antibody	Cell Signaling Technology, 9721	1:400
Caspase-9 (CASP-9)	Q8C3Q9	rabbit polyclonal Caspase-9 antibody	Cell Signaling Technology, 9504	1:600
Apoptosis regulator Bcl-X (Bcl-XL)	Q9QWX2	rabbit monoclonal Bcl-XL antibody	Cell Signaling Technology, 2764	1:600
Apoptosis regulator BAX (BAX)	Q07813	rabbit monoclonal Bax antibody	Cell Signaling Technology, 5023#	1:600
Hepassocin (HPS)	Q71KU9	Rabbit polyclonal HPS antibody	Thermo Fisher Scientific, PA5-30030	1:500
CCAAT/enhancer-binding protein beta (CEBPB)	P28033	Rabbit monoclonal CEBP Beta antibody [E299] - C-terminal	Abcam ab32358	1:1000
Sterol regulatory element-binding protein 1 (SREBP1)	Q9WTN3	mouse monoclonal SREBP1 antibody	Santa Cruz Biotechnology, sc-13551	1:1000
X-box-binding protein 1 (XBP1)	O35426	Rabbit monoclonal XBP1 antibody	Abcam ab220783	1:500

Lamin-A/C (LMNA)	P48678	mouse monoclonal LMNA antibody	Santa Cruz Biotechnology, sc-376248	1:1000
Alpha-tubulin 2 (Tubulin)	P05213	mouse monoclonal tubulin antibody	Abcam ab7291	1:2000
Myc proto-oncogene protein (Myc)	P01108	mouse monoclonal myc antibody	Santa Cruz Biotechnology, sc-40	1:1000
Beta-actin (β-actin)	P60710	mouse monoclonal β-actin antibody	Abcam, ab8226	1:2000
