# Supplementary material for: Protective Role of Hepassocin against Hepatic Endoplasmic Reticulum Stress in Mice
Source: Int J Mol Sci. 2022 Nov 1;23(21):13325. doi: 10.3390/ijms232113325 (PMC9658083; doi:10.3390/ijms232113325)
Supplement: Supplementary file 1 [file ijms-23-13325-s001.zip › Supplementary Table S2.pdf]

Supplementary Table S2: Sequences of primers used for quantitative PCR.

Gene name	Forward (5'-3')	Reverse (5'-3')
Acc1	ATGGGCGGAATGGTCTCTTTC	TGGGGACCTTGTCTTCATCAT
Atf4	GGGTTCTGTCTTCCACTCCA	AAGCAGCAGAGTCAGGCTTTC
Atf6	GACTCACCCATCCGAGTTGTG	CTCCCAGTCTTCATCTGGTCC
Cd36	ATGGGCTGTGATCGGAACTG	GTCTTCCCAATAAGCATGTCTC<br>C
Chop	CTGGAAGCCTGGTATGAGGAT	CAGGGTCAAGAGTAGTGAAGG<br>T
Chop (h)	GGAAACAGAGTGGTCATTCCC	CTGCTTGAGCCGTTCATTCTC
Cpt1	CTCCGCCTGAGCCATGAAG	CACCAGTGATGATGCCATTCT
Derl3	AGGAGGGTTCTTTCCGTGG	ACGGCTCCATACATAGACCAG
Derl3 (h)	AGGTCTGGAGGCTCGTCAC	GCAGTAGCGGAACACGAAGAG
Erdj4	AAATAAAAGCCCTGATGCTGA<br>A	CTATTGGCATCCGAGAGTGTTT
Erdj4 (h)	TCTTAGGTGTGCCAAAATCGG	TGTCAGGGTGGTACTTCATGG
Fasn	GGAGGTGGTGATAGCCGGTAT	TGGGTAATCCATAGAGCCCAG
Fatp2	TCCTCCAAGATGTGCGGTACT	TAGGTGAGCGTCTCGTCTCG
Fatp5	CTACGCTGGCTGCATATAGATG	CCACAAAGGTCTCTGGAGGAT
Grp78	ACTTGGGGACCACCTATTCCT	ATCGCCAATCAGACGCTCC
Grp78 (h)	GAAAGAAGGTTACCCATGCAG<br>T	CAGGCCATAAGCAATAGCAGC
Herpud1	AGCAGCCGGACAACTCTAAT	CTTGGAAAGTCTGCTGGACA
Herpud1 (h)	TGCTGGTTCTAATCGGGGACA	CCAGGGGAAGAAAGGTTCCG
Hrd1	CCTGGGACAACAAGGCTGTA	AGCTTTCTTGAACTGCCTCATG
Hrd1 (h)	CTTCACCGTTTTTCGGGATGA	CCAGGAGGAACATAAGAGAGA<br>CA

HPS	GATTTACAGCTTCGTCCTGG	TCTTGCCTCCAAGGTCAATG
HPS (h)	ATGGCAAAGGTGTTCAGTTTCA	ACAATCTGCATACTGCCTCTTG
Hsp40	TTCGACCGCTATGGAGAGGAA	CACCGAAGAACTCAGCAAACA
Hsp90	TGTTGCGGTACTACACATCTGC	GTCCTTGGTCTCACCTGTGATA
Mttp	CTCTTGGCAGTGCTTTTTCTCT	GAGCTTGTATAGCCGCTCATT
Pparα	AGAGCCCCATCTGTCCTCTC	ACTGGTAGTCTGCAAAACCAAA
Scd1	GTACCGCTGGCACATCAACTT	GGCTAAGACAGTAGCCTTAG
Spliced-Xbp1	CTGAGTCCGAATCAGGTGCAG	GTCCATGGGAAGATGTTCTGG
Srebp1	GGAGCCATGGATTGCACATT	GGCCCGGGAAGTCACTGT
Tbp	AGAACAATCCAGACTAGCAGC A	GGGAACTTCACATCACAGCTC
Tbp (h)	CCACTCACAGACTCTCACAAC	CTGCGGTACAATCCCAGAACT
Unspliced Xbp1	AGCAGCAAGTGGTGGATTTG	GAGTTTTCTCCCGTAAAAGCTG A
Wfs1	CGGGAAGAAACGGACAGAGC	CGTAGGTAGTGTTTGCCCAC
Xbp1	GACAGAGAGTCAAACTAACGT GG	GTCCAGCAGGCAAGAAGGT
Serca	GAGAACGCTCACACAAAGACC	CAATTCGTTGGAGCCCCAT
Stim1	TGACAGGGACTGTACTGAAGATG	TATGCCGAGTCAAGAGAGGAG
Ncx	CGCTGGGGAAGATGACGATG	TGGGACGAAGGCAAACAGAAC
Ip3r	CGTTTTGAGTTTGAAGGCGTTT	CATCTTGCGCCAATTCCCG
Orai	GATCGGCCAGAGTTACTCCG	TGGGTAGTCATGGTCTGTGTC

(h) show that the primer sequence is suitable for detection of human gene.
