# Supplementary figures and images for: Protective Role of Hepassocin against Hepatic Endoplasmic Reticulum Stress in Mice
Source: Int J Mol Sci. 2022 Nov 1;23(21):13325. doi: 10.3390/ijms232113325 (PMC9658083; doi:10.3390/ijms232113325)

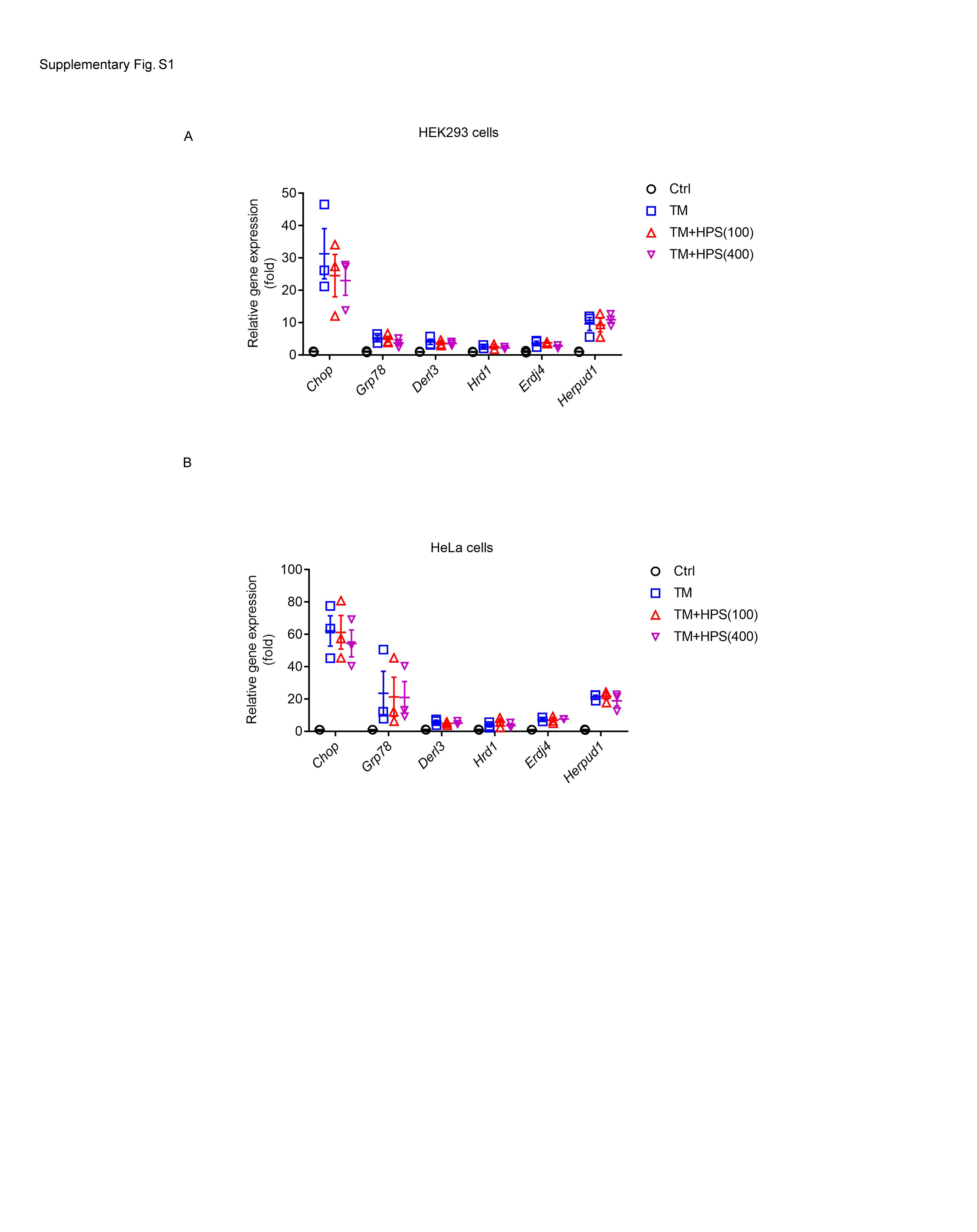

Supplement: Supplementary file 1 [file ijms-23-13325-s001.zip › Supplementary Figure S1.tif]

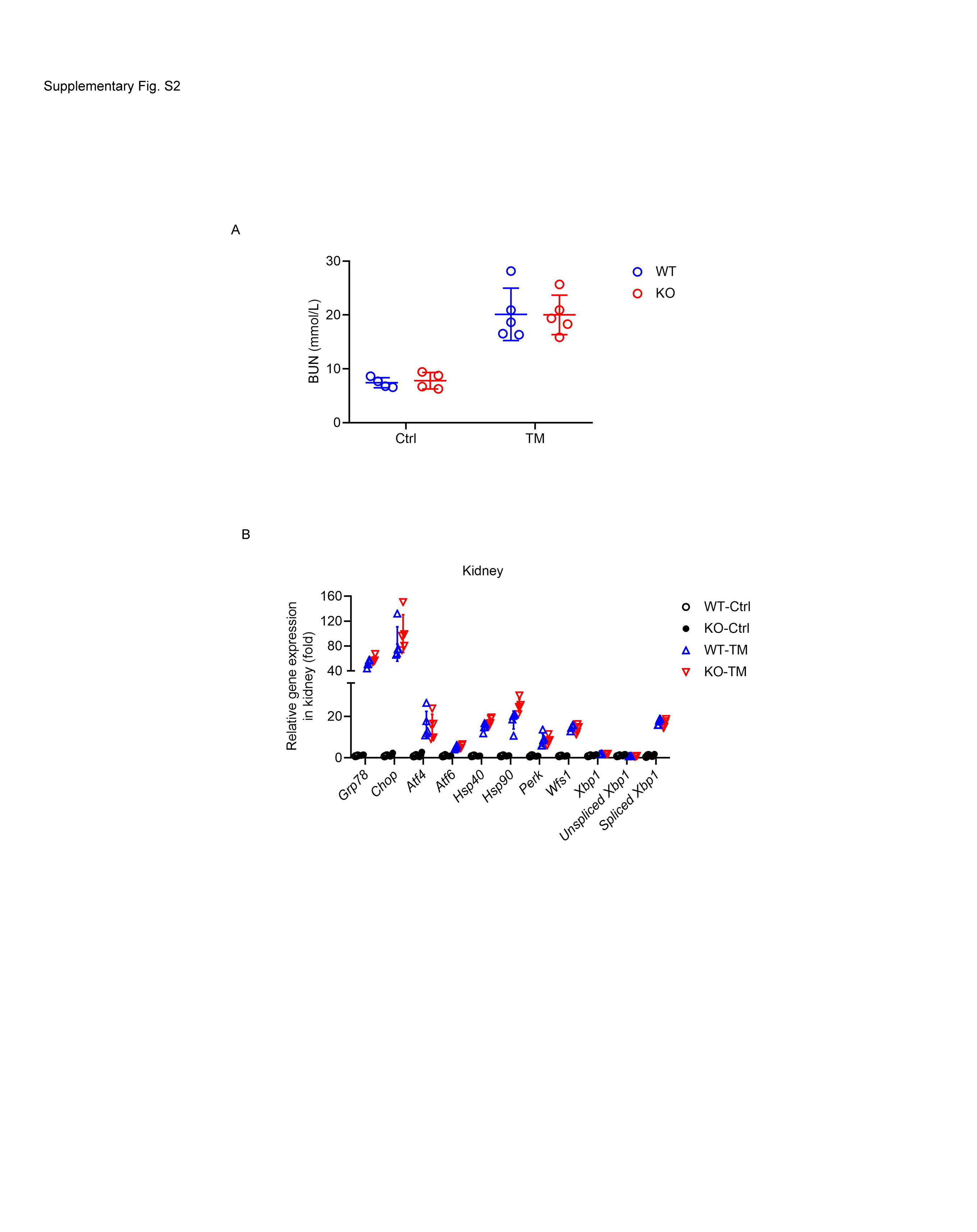

Supplement: Supplementary file 1 [file ijms-23-13325-s001.zip › Supplementary Figure S2.tif]

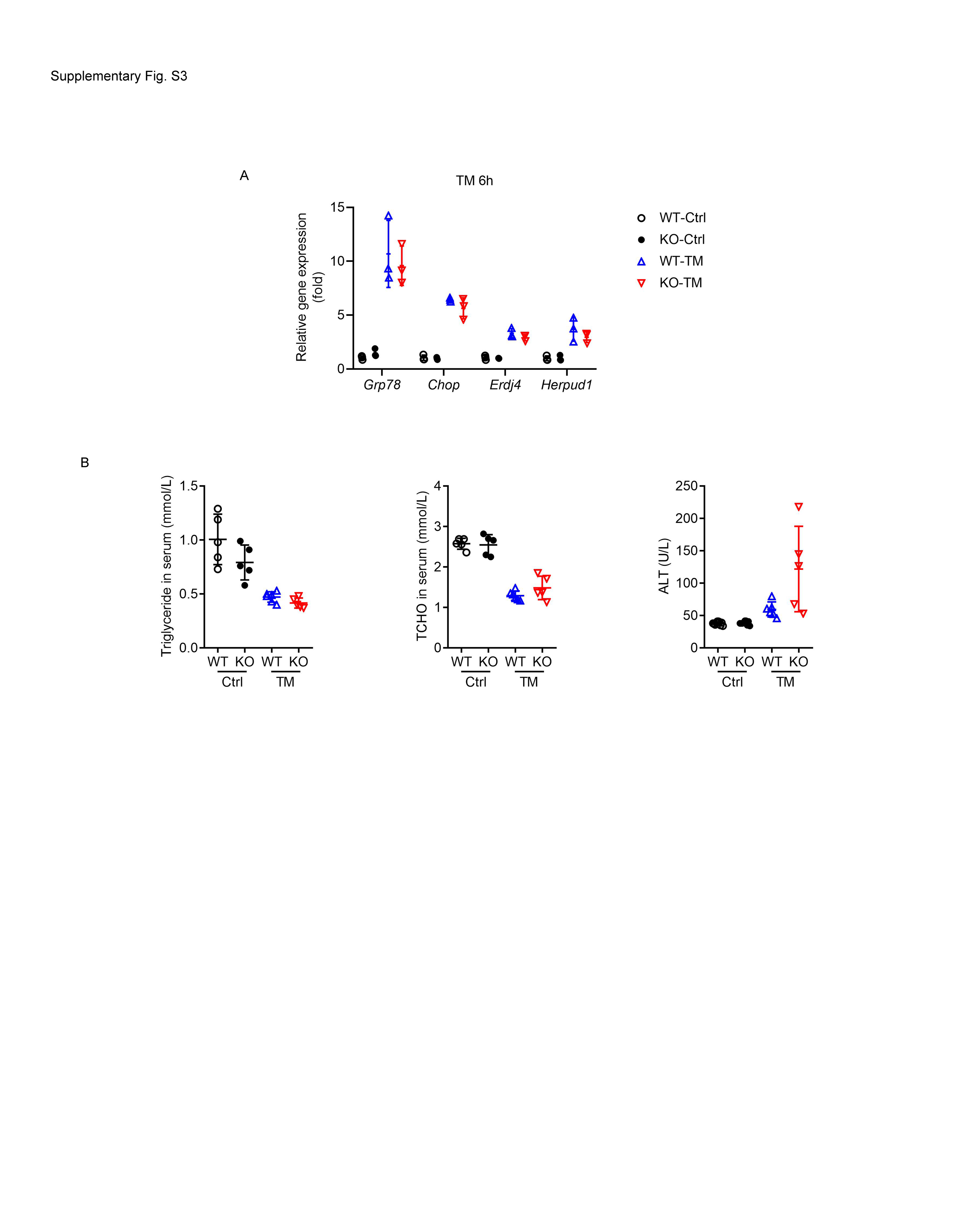

Supplement: Supplementary file 1 [file ijms-23-13325-s001.zip › Supplementary Figure S3.tif]
